# A Questionnaire-Based Ensemble Learning Model to Predict the Diagnosis of Vertigo: Model Development and Validation Study

**DOI:** 10.2196/34126

**Published:** 2022-08-03

**Authors:** Fangzhou Yu, Peixia Wu, Haowen Deng, Jingfang Wu, Shan Sun, Huiqian Yu, Jianming Yang, Xianyang Luo, Jing He, Xiulan Ma, Junxiong Wen, Danhong Qiu, Guohui Nie, Rizhao Liu, Guohua Hu, Tao Chen, Cheng Zhang, Huawei Li

**Affiliations:** 1 Department of Otorhinolaryngology Eye & ENT Hospital Fudan University Shanghai China; 2 Nursing Department Eye & ENT Hospital Fudan University Shanghai China; 3 Department of Information Management and Information Systems Fudan University Shanghai China; 4 State Key Laboratory of Medical Neurobiology and Ministry of Education Frontiers Center for Brain Science Fudan University Shanghai China; 5 National Health Commission Key Laboratory of Hearing Medicine Fudan University Shanghai China; 6 Institutes of Brain Science and the Collaborative Innovation Center for Brain Science Fudan University Shanghai China; 7 Department of Otorhinolaryngology-Head and Neck Surgery The Second Affiliated Hospital of Anhui Medical University Hefei China; 8 Department of Otolaryngology-Head and Neck Surgery The First Affiliated Hospital Medical College, Xiamen University Xiamen China; 9 Department of Otolaryngology-Head and Neck Surgery Shengjing Hospital of China Medical University Shenyang China; 10 Department of Otolaryngology Shanghai Pudong Hospital Shanghai China; 11 Department of Otolaryngology Shenzhen Second People’s Hospital Shenzhen China; 12 Department of Otolaryngology The First Affiliated Hospital of Chongqing Medical University Chongqing China; 13 Institutes of Biomedical Sciences Fudan University Shanghai China

**Keywords:** vestibular disorders, machine learning, diagnostic model, vertigo, ENT, questionnaire

## Abstract

**Background:**

Questionnaires have been used in the past 2 decades to predict the diagnosis of vertigo and assist clinical decision-making. A questionnaire-based machine learning model is expected to improve the efficiency of diagnosis of vestibular disorders.

**Objective:**

This study aims to develop and validate a questionnaire-based machine learning model that predicts the diagnosis of vertigo.

**Methods:**

In this multicenter prospective study, patients presenting with vertigo entered a consecutive cohort at their first visit to the ENT and vertigo clinics of 7 tertiary referral centers from August 2019 to March 2021, with a follow-up period of 2 months. All participants completed a diagnostic questionnaire after eligibility screening. Patients who received only 1 final diagnosis by their treating specialists for their primary complaint were included in model development and validation. The data of patients enrolled before February 1, 2021 were used for modeling and cross-validation, while patients enrolled afterward entered external validation.

**Results:**

A total of 1693 patients were enrolled, with a response rate of 96.2% (1693/1760). The median age was 51 (IQR 38-61) years, with 991 (58.5%) females; 1041 (61.5%) patients received the final diagnosis during the study period. Among them, 928 (54.8%) patients were included in model development and validation, and 113 (6.7%) patients who enrolled later were used as a test set for external validation. They were classified into 5 diagnostic categories. We compared 9 candidate machine learning methods, and the recalibrated model of light gradient boosting machine achieved the best performance, with an area under the curve of 0.937 (95% CI 0.917-0.962) in cross-validation and 0.954 (95% CI 0.944-0.967) in external validation.

**Conclusions:**

The questionnaire-based light gradient boosting machine was able to predict common vestibular disorders and assist decision-making in ENT and vertigo clinics. Further studies with a larger sample size and the participation of neurologists will help assess the generalization and robustness of this machine learning method.

## Introduction

Dizziness and vertigo are the major complaints of patients with vestibular disorders, with an estimated lifetime prevalence of dizziness (including vertigo) of 15%-35% [1]. Dizziness and vertigo are incapacitating and considerably impact patients’ quality of life. These conditions often lead to activity restriction and are closely associated with psychiatric disorders such as anxiety, phobic, and somatoform disorders [1-3]. Patients with dizziness and vertigo are also at a higher risk of falls and fall-related injuries, especially older people [4]. However, the diagnosis of vestibular disorders is challenging and time-consuming. It involves a variety of vestibular and neurological causes and complex pathological processes, leading to misdiagnosis and potentially widespread overuse of imaging among vertiginous patients [5-8]. Consequent delays in diagnosis can worsen the functional and psychological consequences of the disease.

The application of artificial intelligence in diagnosing dizziness and vertigo dates back more than 30 years. Expert systems such as *Vertigo* [9], *Carrusel* [10], and *One* [11] consist of knowledge bases with fixed diagnostic rules. They infer through nonadaptive algorithms that were unable to learn from patients’ data. Different machine learning algorithms, including genetic algorithms, neural networks, Bayesian methods, k-nearest neighbors, and support vector machines, have also been employed to analyze patient data from *One* [12-16]. The predictive accuracy was 90%-97% for 6 common otoneurologic diagnoses and 76.8%-82.4% for 9 diagnostic categories. *EMBalance* is a comprehensive platform that was launched in 2015 to assist the diagnosis, treatment, and evolution of balance disorders by using ensemble learning methods based on decision trees (Adaptive Boosting) [17,18]. There has been a shift from pure knowledge-driven to data-driven methodology in computer-aided diagnosis of vestibular disorders.

Except *Vertigo*, all of the models mentioned above are based on patients’ medical history and examinations combined with necessary tests, while in practice, patient history alone provides important clues to possible diagnosis and further evaluation [19]. Numerous questionnaires for dizziness and vertigo have emerged during the past 2 decades to assist the clinical diagnosis of vestibular disorders [20-27]. Most of these studies used simple statistical models, typically logistic regression, validated with the same data as modeling [26-28]. Few studies have tried to apply machine learning algorithms. However, the accuracy of these models was not as good as that of simple statistical models owing to small data sets or inappropriate choice of modeling data [29,30].

This study is part of the Otogenic Vertigo Artificial Intelligence Research (OVerAIR) study, in which the overarching purpose is to build a comprehensive platform that integrates diagnosis, treatment, rehabilitation, and follow-up in a cohort of patients with otogenic vertigo by using artificial intelligence. The specific aims of this study include developing and verifying a diagnostic platform for vertigo and assisting clinical decision-making by using machine learning techniques and further exploring the effectiveness and clinical utility of the proposed platform.

## Methods

### Study Design

Patients presenting with a new complaint of vertigo or dizziness according to the classification of vestibular symptoms by the Barany Society [31] were enrolled consecutively from the ENT and vertigo clinics of Eye & ENT Hospital of Fudan University, The Second Hospital of Anhui Medical University, The First Affiliated Hospital of Xiamen University, Shengjing Hospital of China Medical University, Shanghai Pudong Hospital, Shenzhen Second People’s Hospital, and The First Affiliated Hospital of Chongqing Medical University from August 2019 through March 2021. At their first interview with an ENT specialist, patients completed the electronic version of the questionnaire via a tablet or smartphone after giving informed consent. Those who were unable to read and complete the questionnaire by themselves answered the questions read by the researchers. We did not interfere with the normal medical procedures of the patients. Patients were scheduled for a next visit as the specialist considered necessary; therefore, they did not stick to a fixed follow-up time.

### Ethics Approval

This study was approved by the Institutional Review Boards of all participating centers (approval 2019091). This study followed the Transparent Reporting of a Multivariable Prediction Model for Individual Prognosis or Diagnosis reporting guidelines [32].

### Outcomes

Each patient went through routine history collection followed by complete otoneurological examinations, and further workup (ie, pure tone audiometry, vestibular testing, computed tomography, and magnetic resonance imaging) was prescribed when necessary. The clinical diagnosis given by ENT specialists with more than 5 years of clinical experience who were blinded to questionnaire responses was used as the reference diagnosis. The reference diagnostic standards include practice guidelines for benign paroxysmal positional vertigo (BPPV) by the American Academy of Otolaryngology-Head and Neck Surgery [33] and diagnostic criteria for vestibular disorders (including vestibular migraine [34], Meniere disease [35], persistent postural-perceptual dizziness [36], vestibular paroxysmia [37], and bilateral vestibulopathy [38]) by the Barany society. Patients with typical clinical features who did not meet the criteria of definite diagnosis were given probable diagnosis. Patients without a specific diagnosis within 2 months or who stopped coming for visits before reaching a final diagnosis were labeled undetermined.

### Questionnaire Development

The diagnostic questionnaire was developed through an iterative process that mainly consisted of the following 3 stages.

1. Focus group and panel meeting: First, a focus group discussion and 3 follow-up panel meetings were convened to identify the commonly seen peripheral vestibular disorders in ENT clinics. In this process, 16 disorders were identified and the featured manifestations of each disorder were listed. The literature of diagnostic or practice guidelines for each disorder was searched and the pertinent ones were carefully reviewed. After that, the initial questionnaire composed of 43 items was drafted.
2. Patient interview: Fifteen patients who presented with vertigo in our ENT clinic were interviewed for the understandability and easiness of filling out the questionnaire. Two patients reported that it was too long and time-consuming. Another 3 complained of being asked too many questions such as heart disease and medication taken, which seemed unrelated to their vertigo condition. At this stage, the wording of the questionnaire was thoroughly simplified and 6 questions were deleted.
3. Expert group meeting: At a national conference, 12 experts (from ENT, neurology, vestibular examination, and rehabilitation) were invited to evaluate the suitability and clarity of the questionnaire, and they put forward suggestions for further revision. During this process, the items were reordered and some were combined or omitted.

### Statistical Analysis

We compared 9 candidate machine learning methods to screen for the one with the best performance. Five non–ensemble learning algorithms were considered, namely, decision tree [39], ridge regression [40], logistic regression (with L2-regularization) [41], support vector classification [42], and support vector classification with stochastic gradient descent [43]. Ensemble learning refers to a general meta approach that strategically improves predictive performance by combining the predictions from multiple models. Four of the ensemble learning methods were implemented, namely, random forest [44], Adaptive Boosting [45], gradient boosting decision tree [46], and light gradient boosting machine (LGBM) [47]. We took bootstrapped cross-validation that randomly sampled data into train and validation sets by 7:3, which were repeated 100 times with replacement [48]. Models were trained on the training set and evaluated based on the prediction performance on the validation set. The best model was selected and tuned based on the average prediction performance over the 100 validation set. The area under the curve (AUC) was used to evaluate the performance of the models. In multiclass prediction, sensitivity, specificity, likelihood ratio, and AUC were calculated through a one-vs-rest scheme (microaverage). Then, recalibration was performed using calibration curves [49] and Brier scores [50] to adjust the difference between the predicted probability and observed proportion of each diagnostic category. External validation was performed using the data of the newest patients in the cohort (enrolled during the last 2 months), which constituted the test set. The 95% CIs of all the metrics were calculated through bootstrapping.

The missing values of Boolean variables were imputed with False in the main results, and sensitivity analysis was conducted by comparing different imputation strategies (ie, without imputation or imputation with True). All machine learning algorithms were implemented in Python, and the code is available in online resources. Hyperparameters are set to default according to the state-of-art machine learning package: sklearn.

### Robustness and Sample Size Analysis

As a data-driven prediction approach for boosting clinical diagnosis, it is necessary to verify that the number of samples is enough for model development and validation. Following Riley [51] and Riley et al [52], we quantified the sufficiency of sample size in terms of the global shrinkage factor and the minimal number of samples. The criterion of enough sample size is to ensure a shrinkage factor >0.9. Further, given the acceptable shrinkage factor (eg, 0.9), the necessary size of the samples to develop a prediction model can be estimated based on the Cox-Snell ratio of explained variance.

Further, the increased flexibility of modern techniques implies that larger sample sizes may be required for reliable estimation compared with classical methods such as logistic regression. Thus, we followed the approach of van der Ploeg et al [53] to evaluate our best model LGBM’s sensitivity on sample size. The training set is of different sizes and subsampled from the development set. Each training set size is repeated 30 times to eliminate randomness, while the average AUC measures the performance on the test set.

### Important Variables

To measure the importance of variables, we first evaluated multivariate feature importance according to information gains in cross-validation and selected the top 20 important variables. Then, to figure out feature importance in individual diagnostic categories, each selected variable was used to predict the 5 diagnostic categories independently, and univariate variable importance was measured in terms of AUC.

## Results

### Overview of the Diagnostic Questionnaire

The final questionnaire consists of 23 items that incorporated branching logic. The full version of the questionnaire is available in Multimedia Appendix 1. The contents of the items are shown in Textbox 1.

**Items in the diagnostic questionnaire.**

- One question on the characteristic of the symptom: was the head spinning or not? If not, then the kind of dizziness needs to be specified (heavy/muddled head, staggering, or other)
- Three questions on the frequency, duration, and duration it has been since the first vertigo attack
- One question on the condition of hearing loss, that is, which side and how it changes
- Three questions on the condition of tinnitus, aural fullness, and earache, that is, which side and whether it changes before and after the attack should be specified (aggravate before/during the attack, relieve after the attack)
- One question on the presence of headache, specifically the time of headache attack and relevant family history
- One question on accompanied photophobia or phonophobia
- One question on unsteadiness during, after, or without vertigo attacks
- One question on whether symptoms worsen when standing or walking
- Two questions on the condition of fall, consciousness state, and whether there was incontinence during the attack
- Five questions on the triggering factors of vertigo, that is, lying down, turning over, getting up quickly, holding breath, loud stimulation, in some special scenes, special foods or smells, fatigue, insomnia, and getting angry
- One question on whether it is cervical vertigo, that is, upper limb numbness and pain or neck pain
- One question on prodrome, that is, cold, fever, and diarrhea before onset
- One question on the medical history of otological disorders, that is, otorrhea, otitis media, ear surgery
- One question on head and neck trauma and surgery history

### Demographic Characteristics of the Participants

A prospective cohort of 1693 patients was enrolled from the ENT and vertigo clinics of 7 participating centers (Table 1). The response rate was 96.2% (1693/1760, 67 declined participation). Of the 1693 enrolled patients, 1041 (61.5%) received 1 final diagnosis by the treating specialists, 14 (0.8%) had more than one diagnosis, 145 (8.6%) had a probable diagnosis, while the other 493 (29.1%) did not receive the final diagnosis within 2 months. The final diagnoses were found to be unevenly distributed. The most common diagnoses were BPPV, vestibular migraine, sudden sensorineural hearing loss with vestibular dysfunction (SSNHL-V), and Meniere disease. Since only patients with 1 final diagnosis were included in the model development and validation, 1041 patients (median age 50 [IQR 38-61] years, 608 [58.4%] females) in the 5 diagnostic categories were included in the model development and validation. Less frequent diagnoses with no more than 20 cases were labeled as “others” for the moment because there were not sufficient cases for them to form separate categories.

**Figure 1:**
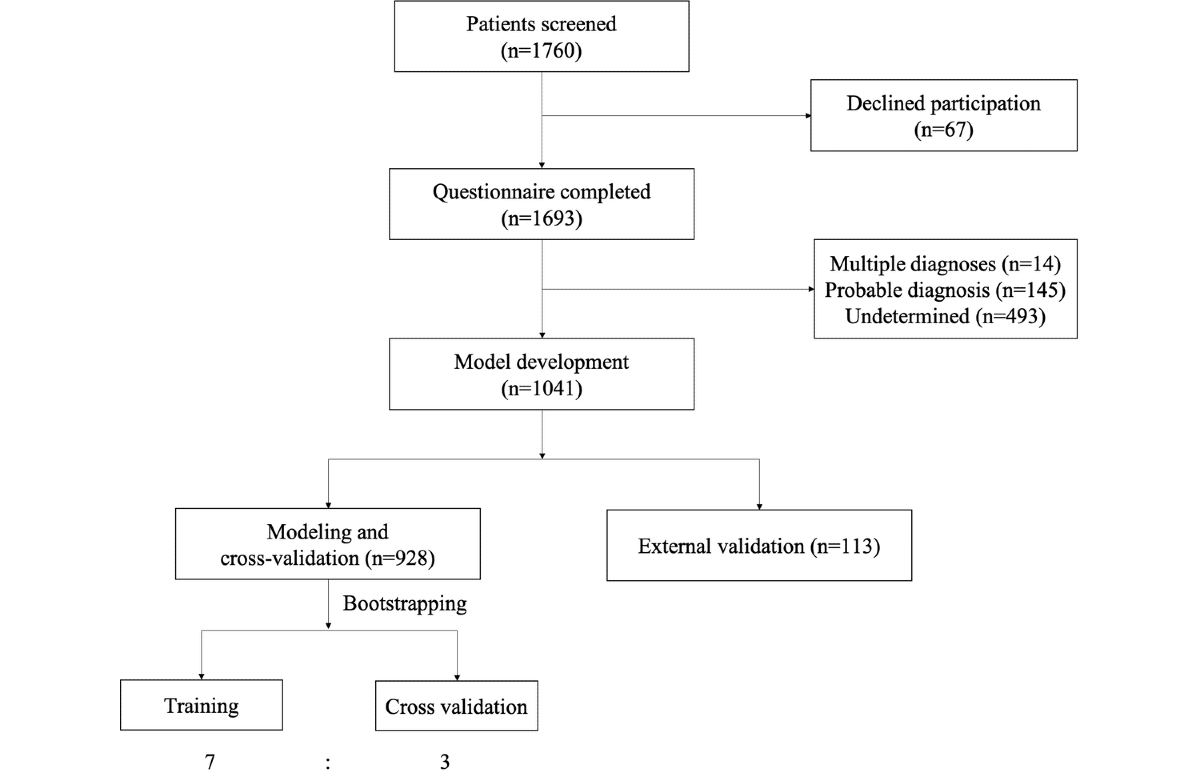
Patients with a new vertigo or dizziness complaint were screened between August 2019 and March 2021. Diagnoses were recorded within 2 months of follow-up.

Of the 1041 patients, 928 were classified into the training set (for modeling and cross-validation) and 113 were included in the test set (Table 2). Figure 1 shows the study flowchart. The details of the training set and test set are described in Table 2.

**Table 1:** Demographic characteristics of the participants (N=1693).

Characteristic		Value
Age (years), median (IQR)		51 (38-61)
**Sex, n (%)**		
	Female	991 (58.5)
	Male	702 (41.6)
**Diagnoses, n (%)**		
	Benign paroxysmal positional vertigo	398 (23.5)
	Vestibular migraine	203 (12)
	Meniere disease	194 (11.5)
	Sudden sensorineural hearing loss with vestibular dysfunction	173 (10.2)
	Others^a^	73 (4.3)
	Multiple diagnosis	14 (0.8)
	Probable diagnosis	145 (8.6)
	Undetermined	493 (29.1)

^a^This category included vestibular neuritis, persistent postural-perceptual dizziness, psychogenic dizziness, delayed endolymphatic hydrops, vestibular paroxysmia, cervicogenic vertigo, acoustic neuroma, presbyvestibulopathy, light cupula, Ramsay-Hunt syndrome, labyrinthine fistula, and superior semicircular canal dehiscence syndrome.

**Table 2:** Characteristics of the training data set and test set.

Characteristic		Training set (n=928)	Test set (n=113)
Age (years), median (IQR)		50 (37-60)	53 (41-63)
**Sex, n (%)**			
	Female	536 (57.8)	72 (63.7)
	Male	392 (42.2)	41 (36.3)
**Diagnoses, n (%)**			
	Benign paroxysmal positional vertigo	348 (37.5)	50 (44.2)
	Vestibular migraine	182 (19.6)	21 (18.6)
	Meniere disease	168 (18.1)	26 (23)
	Sudden sensorineural hearing loss with vestibular dysfunction	164 (17.6)	9 (8)
	Others^a^	66 (7.1)	7 (6.2)

^a^This category included vestibular neuritis, persistent postural-perceptual dizziness, psychogenic dizziness, delayed endolymphatic hydrops, vestibular paroxysmia, cervicogenic vertigo, acoustic neuroma, presbyvestibulopathy, light cupula, Ramsay-Hunt syndrome, labyrinthine fistula, and superior semicircular canal dehiscence syndrome.

### Development and Validation of the Model

The LGBM model had the highest AUC of 0.937 (95% CI 0.917-0.962) and the lowest Brier score of 0.057 (95% CI 0.049-0.068) among the 9 models in cross-validation (Table 3). Therefore, it was recalibrated and used as the final predictive model.

For sensitivity analysis, when imputing the missing value with mode (the most frequent label), the AUC and Brier score of all 9 methods dropped (Table 4). Note that LGBM does not rely on imputation methods; therefore, it can directly utilize the information from *missing* to achieve a better prediction performance. LGBM without imputation performs as well as the recalibrated LGBM (imputed with 0), which verifies the robustness of our method. Ensemble learning methods performed better than non–ensemble learning methods except logistic regression with LASSO in cross-validation, indicating that the introduction of ensemble learning in vertigo diagnosis is effective across specific ensemble approaches. Further, LGBM performs better than other methods in AUC and Brier scores.

**Figure 2:**
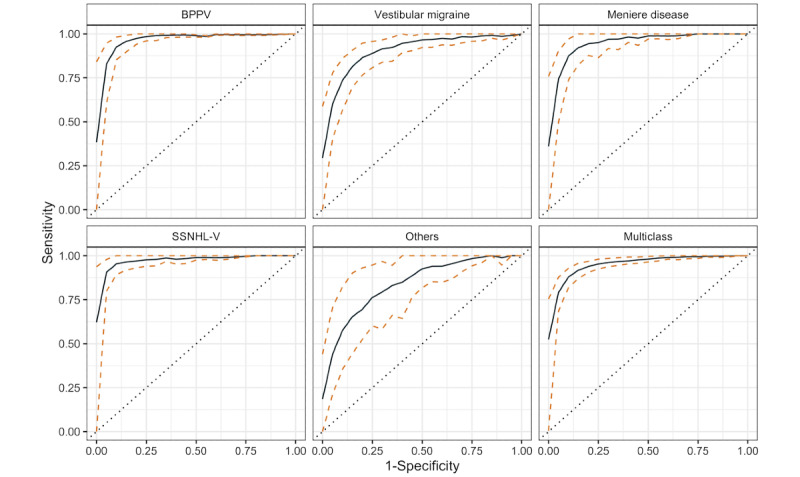
The receiver operating characteristic curves (solid lines) with 95% CI (between 2 dashed lines) for each diagnostic category. The performance of each diagnostic category was evaluated through one-vs-rest scheme. BPPV: benign paroxysmal positional vertigo; SSNHL-V: sudden sensorineural hearing loss with vertigo.

The receiver operating characteristic curves of the recalibrated LGBM model in cross-validation are shown in Figure 2. Table 5 presents the AUC, sensitivity, specificity, likelihood ratios, and accuracy in different diagnostic categories in both cross and external validation. The model made highly accurate prediction for SSNHL-V (AUC>0.98, positive likelihood ratio [+LR]>20, negative likelihood ratio [–LR]<0.05), accurate prediction for BPPV and Meniere disease (AUC>0.95, sensitivity>0.8, specificity>0.9, accuracy>0.9, +LR>10, –LR<0.2), and showed fair discriminative ability for vestibular migraine (AUC 0.9, 95% CI 0.87-0.92). The prediction of other diagnoses was unstable owing to the limited sample size and great heterogeneity in this category, with an AUC ranging from 0.771 to 0.929 in cross-validation and 0.879 to 0.957 in external validation.

**Figure 3:**
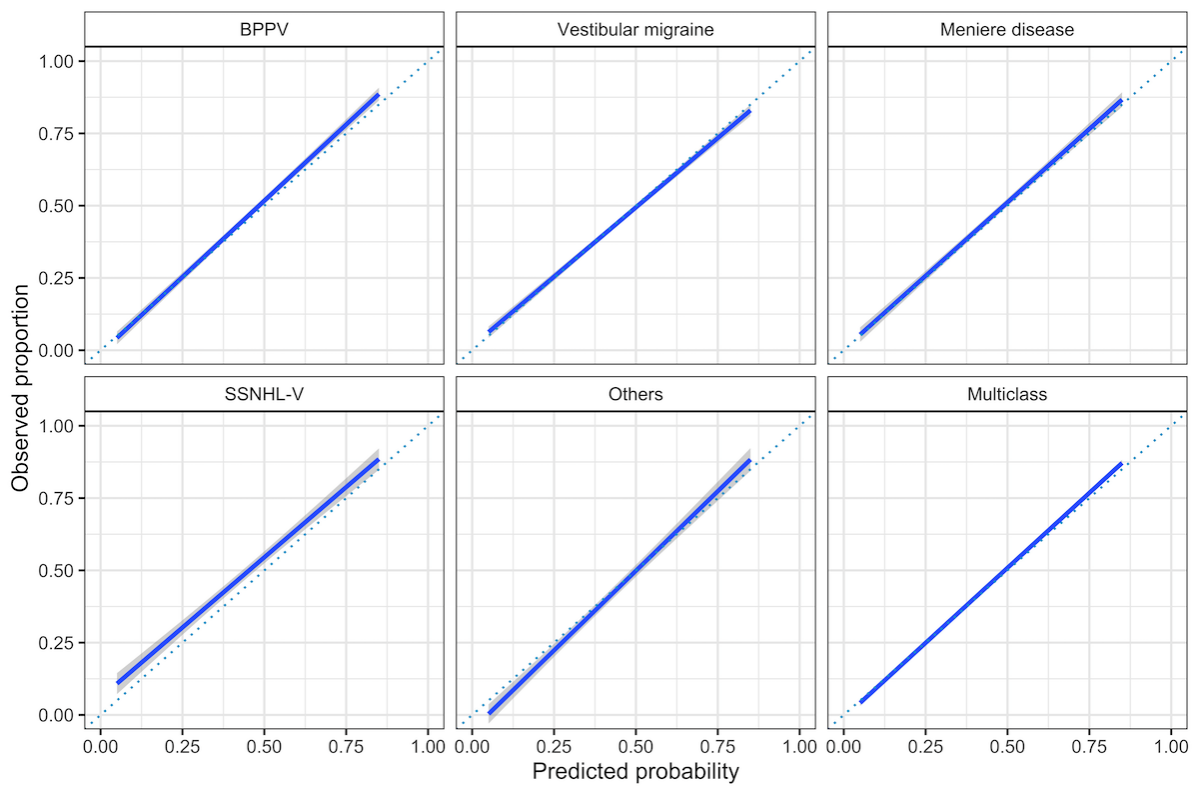
Calibration curves (blue solid lines) with pointwise 95% confidence limits (grey ribbon) on the validation data based on recalibrated light gradient boosting machine model. BPPV: benign paroxysmal positional vertigo; SSNHL-V: sudden sensorineural hearing loss with vertigo.

Calibration curves in cross-validation (Figure 3) properly estimated the probability of Meniere disease and vestibular migraine and slightly underestimated the probability of SSNHL-V and BPPV. The predictions for other diagnoses were relatively conservative, as it was less likely to give probabilities close to 0 or 1. The Brier score was 0.058 (95% CI 0.049-0.068) in cross-validation, which suggested that the predicted probabilities fitted well with the actual proportions of the diagnoses. We also applied our methods to the external data set. The results indicated that the selected best model, LGBM, was of generalization ability in predicting vertigo diagnosis, achieving an AUC of 0.958 (95% CI 0.951-0.969). Meanwhile, LGBM also performed better than the second-best method, logistic regression, which achieved an AUC of 0.939 (95% CI 0.925-0.956) in external validation. The multivariable feature importance in terms of information gain is shown in Table 6.

**Figure 4:**
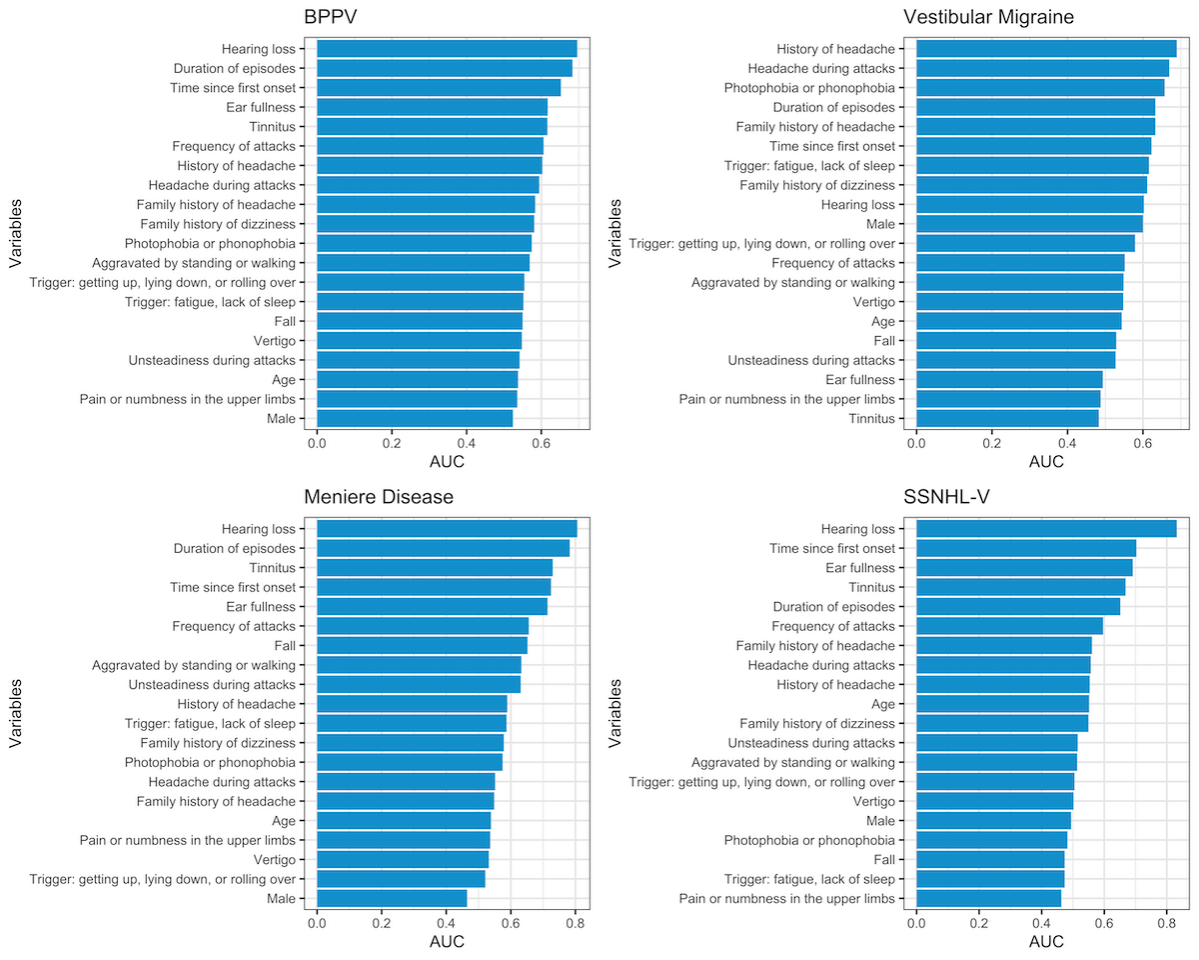
Area under the curve in univariate prediction was used as the estimate of variable importance. AUC: area under the curve; BPPV: benign paroxysmal positional vertigo; SSNHL-V: sudden sensorineural hearing loss with vertigo.

The analysis of the global shrinkage factor of each diagnostic category and sensitivity analysis results indicated that the sample size of this study is sufficient for model development. See Multimedia Appendix 2 for more details of sample size analysis. Then, to figure out feature importance in individual diagnostic categories, each of the top 20 contributing variables in Table 6 was used to predict the 5 diagnostic categories independently, and univariate variable importance was measured in terms of AUC (Figure 4).

**Table 3:** The prediction performance of candidate algorithms.

Method		Area under the curve (95% CI)	Brier score (95% CI)
**Non–ensemble learning**			
	Decision tree	0.765 (0.726-0.798)	0.125 (0.104-0.146)
	Ridge regression	0.803 (0.780-0.831)	0.087 (0.071-0.104)
	Logistic regression	0.928 (0.907-0.956)	0.060 (0.051-0.069)
	Support vector classification	0.501 (0.499-0.505)	0.239 (0.220-0.258)
	Stochastic gradient descent	0.733 (0.611-0.824)	0.141 (0.083-0.254)
**Ensemble learning**			
	Random forest	0.924 (0.900-0.949)	0.063 (0.056-0.070)
	Adaptive Boosting	0.851 (0.793-0.901)	0.148 (0.144-0.151)
	Gradient boosting decision tree	0.925 (0.902-0.951)	0.064 (0.053-0.076)
	Light gradient boosting machine	0.935 (0.913-0.960)	0.057 (0.047-0.067)
	Recalibrated light gradient boosting machine	0.937 **(**0.917-0.962)	0.058 (0.049-0.068)

**Table 4:** Performance of different algorithms while imputing missing data with mode.

Method		Area under the curve (95% CI)	Brier score (95% CI)
**Non–ensemble learning**			
	Decision tree	0.746 (0.690-0.791)	0.137 (0.114-0.169)
	Ridge regression	0.788 (0.733-0.817)	0.096 (0.076-0.121)
	Logistic regression	0.921 (0.900-0.943)	0.067 (0.057-0.082)
	Support vector classification	0.500 (0.500-0.500)	0.240 (0.222-0.258)
	Stochastic gradient descent	0.727 (0.578-0.819)	0.148 (0.090-0.251)
**Ensemble learning**			
	Random forest	0.919 (0.896-0.939)	0.068 (0.061-0.078)
	Adaptive Boosting	0.833 (0.741-0.887)	0.148 (0.143-0.156)
	Gradient boosting decision tree	0.915 (0.888-0.935)	0.073 (0.059-0.093)
	Light gradient boosting machine	0.929 (0.906-0.950)	0.062 (0.055-0.072)
	Light gradient boosting machine (without imputation)	0.935 (0.916-0.956)	0.057 (0.049-0.065)

**Table 5:** Predictive ability in different diagnostic categories.

		AUC^a^ (95% CI)	Sensitivity (95% CI)	Specificity (95% CI)	+LR^b^ (95% CI)	–LR^c^ (95% CI)	Accuracy (95% CI)
** Benign paroxysmal positional vertigo **							
	CV^d^	0.97 (0.96-0.99)	0.94 (0.87-0.99)	0.92 (0.85-0.97)	13.23 (6.55-29.3)	0.07 (0.01-0.14)	0.92 (0.89-0.95)
	EV^e^	0.98 (0.97-0.99)	0.97 (0.92-1)	0.90 (0.83-0.94)	10.23 (5.88-17.92)	0.04 (0-0.09)	0.93 (0.90-0.96)
** Vestibular migraine **							
	CV	0.91 (0.87-0.94)	0.86 (0.76-0.95)	0.85 (0.74-0.95)	6.58 (3.56-13.93)	0.17 (0.07-0.27)	0.85 (0.78-0.92)
	EV	0.9 (0.87-0.92)	0.66 (0.52-0.76)	0.90 (0.85-0.96)	7.38 (4.71-12.05)	0.38 (0.26-0.51)	0.86 (0.82-0.88)
**Sudden sensorineural hearing loss with vertigo**							
	CV	0.99 (0.97-1)	0.95 (0.88-1)	0.95 (0.90-0.99)	25.07 (9.39-67.93)	0.05 (0-0.12)	0.95 (0.91-0.98)
	EV	1.00 (1.00-1.00)	1.00 (1.00-1.00)	0.98 (0.97-1.00)	Inf^f^ (34.67-Inf)	0.00 (0.00-0.00)	0.98 (0.97-1)
**Meniere disease**							
	CV	0.96 (0.93-0.98)	0.92 (0.81-1)	0.90 (0.82-0.96)	10.79 (5.28-22)	0.09 (0-0.21)	0.90 (0.84-0.95)
	EV	0.97 (0.97-0.98)	0.82 (0.69-0.88)	0.98 (0.95-0.99)	Inf (18.4-Inf)	0.19 (0.12-0.31)	0.94 (0.91-0.96)
**Others**							
	CV	0.86 (0.77-0.93)	0.83 (0.66-1)	0.78 (0.55-0.93)	4.44 (2.10-9.77)	0.21 (0-0.44)	0.78 (0.57-0.91)
	EV	0.92 (0.88-0.96)	0.74 (0.50-0.86)	0.90 (0.85-0.94)	7.59 (5.05-12.02)	0.38 (0.26-0.51)	0.89 (0.85-0.93)

^a^AUC: area under the curve.

^b^+LR: positive likelihood ratio.

^c^–LR: negative likelihood ratio.

^d^CV: cross-validation.

^e^EV: external validation.

^f^Inf: Positive likelihood ratio was infinity because specificity was 1.

**Table 6:** Multivariable feature importance in light gradient boosting machine model.

Variable	Feature importance
Sudden hearing loss	1039.8
Duration of episodes	912.3
Hearing loss	694.8
Time since first onset	468.1
Trigger: getting up, lying down, or rolling over	358.0
Age	255.6
History of headache	250.6
Frequency of attacks	221.4
Fluctuating hearing loss	186.3
Photophobia or phonophobia	185.7
Time since first hearing loss	183.7
Recurring symptoms	155.9
Tinnitus	135.5
Ear fullness	135.4
Headache during attacks	117.7
Aggravated by standing or walking	80.4
Trigger: fatigue, lack of sleep	69.7
Vertigo	65.0
Pain or numbness in the upper limbs	62.4
Unsteadiness during attacks	59.5
Family history of headache	54.2
Male	54.1
Fall	47.3
Loss of consciousness, incontinence	44.6
Tinnitus: aggravated before an attack, alleviated after an attack	36.7
Trigger: visual stimuli	31.0
Trigger: sound and pressure	23.0
Unsteadiness: after first onset	22.4
Prodrome: cold, fever, vomiting, or diarrhea	22.0
Family history of dizziness	17.4
Trigger: certain foods	15.9
Otalgia	11.6
Conscious when falling	9.8
History of otitis media or ear surgery	7.2
Tinnitus: worsen during vertigo	4.5
Fluctuating: gradually worsen	0.0
Unsteadiness between attackss	0.0
Recent history of head and neck trauma or surgery	0.0

## Discussion

### Principal Findings

In this multicenter prospective cohort study, a questionnaire was developed to diagnose vertigo, and an LGBM model was developed using patients’ historical data collected through the questionnaire. This is, to our knowledge, the first questionnaire-based machine learning model to predict multiple diagnoses of vertigo. Because all the patients in this study were from ENT and vertigo clinics, the distribution of diagnoses differs from that in previous studies conducted in neurology and balance clinics [19-21,26]. There was a much higher prevalence of SSNHL-V (173/1693, 10.2%) and a lower prevalence of vestibular neuritis (22/1693, 1.3%) in our study.

Our model outperformed previously reported questionnaire-based statistical models in predicting common vestibular diagnoses [20,21,26]. A possible explanation is that machine learning methods are better at dealing with potentially nonlinear relationships and overfitting. Additionally, given the subjectivity of patient-reported historical information, data-driven models are better fits in questionnaire-based prediction than knowledge-driven models [9,11,54,55]. Compared with previous machine learning diagnostic systems that used comprehensive patient history data, physical examination, and laboratory tests, our questionnaire-based diagnostic model has its merits [13-17]. First, medical history provides important clues to the cause of vertigo, based on which the doctor will try to confirm or exclude a presumptive diagnosis. Therefore, a questionnaire-based diagnostic tool can provide early decision support according to patient history and help reduce unnecessary workup. Further, since questionnaire data come directly from patients, the model’s performance does not rely on the accurate interpretation of patient history by professionals. Besides, considering the limited accessibility of specific tests (eg, pure tone audiometry, caloric test, video head impulse test), a questionnaire requiring no special equipment is suitable across different clinical settings. However, a questionnaire-based diagnostic model also has intrinsic limitations. Patient-reported medical history can be imprecise because it can be easily affected by recall bias, misinterpretation, emotional state of the patients, and other subjective factors. Meanwhile, for patients with only nonspecific symptoms, physical examination and laboratory testing are more important diagnostic tools. Patient history should always be combined with objective evidence to make a more reliable diagnosis. Therefore, it is necessary to introduce physical examination and laboratory test results into the system in the future to make a comprehensive stepwise diagnostic prediction.

### Limitations

This study had the following limitations. The uneven distribution of diagnoses made it difficult for the model to give accurate predictions of rare diagnoses. In order to reduce potential noise, we included only patients with 1 final diagnosis in modeling. The exclusion of patients with undetermined diagnosis was a potential source of bias. There were several reasons that these patients did not receive a specific diagnosis. In some cases, patients with BPPV might experience spontaneous remission while waiting for the scheduled positional test and treatment (1-2 weeks later), which also explains the relatively low prevalence of BPPV in our cohort than that in other ENT clinics [56]. The exclusion of these patients could reduce noise and improve model performance. Besides, some patients only experienced transient symptoms without observable structural, functional, or psychological changes; therefore, no specific diagnosis was given. Moreover, while a majority of patients completed all the necessary examinations within the follow-up, it was also possible that some rare causes were not determined within 2 months, possibly adding to the imbalance of data. Nevertheless, as the cohort expands, more patients with rare diagnoses will be included, which will enable the model to predict rare diagnoses with higher accuracy. We can also manage the influence of imbalanced data during modeling. Meanwhile, the observed AUC in external validation was higher than that in cross-validation, which could be accounted for by the relatively small sample size of the test set. More participants with definite diagnosis are needed for providing further validation. Finally, since this study was conducted in the ENT and vertigo clinic of tertiary centers, the predictive power of the model is yet to be verified in different clinical settings.

### Conclusion

This study presents the first questionnaire-based machine learning model for the prediction of common vestibular disorders. The model achieved strong predictive ability for BPPV, vestibular migraine, Meniere disease, and SSNHL-V by using an ensemble learning method LGBM. As part of the OVerAIR platform, it can be used to assist clinical decision-making in ENT clinics and help with the remote diagnosis of BPPV. We have also been working on a smartphone app that integrates the questionnaire with referral, follow-up, treatment, and rehabilitation to improve the health outcomes of patients with vertigo. The next phase of the OVerAIR study will involve the participation of neurologists, which is expected to improve the model’s predictive ability for central vertigo and help assess its generalization and robustness.

## Appendix

Multimedia Appendix 1 The final version of the diagnostic questionnaire for vertigo applied in this study.

Multimedia Appendix 2 Sample size analysis.

## Abbreviations

AUC: area under the curve
BPPV: benign paroxysmal positional vertigo
LGBM: light gradient boosting machine
OVerAIR: Otogenic Vertigo Artificial Intelligence Research
SSNHL-V: sudden sensorineural hearing loss with vestibular dysfunction
