# Supplementary material for: A Questionnaire-Based Ensemble Learning Model to Predict the Diagnosis of Vertigo: Model Development and Validation Study
Source: J Med Internet Res. 2022 Aug 3;24(8):e34126. doi: 10.2196/34126 (PMC9386585; doi:10.2196/34126)
Supplement: Multimedia Appendix 1 [file jmir_v24i8e34126_app1.docx]

**Multimedia Appendix 1. Diagnostic Questionnaire for Vertigo**

1. Did you feel the world revolving, your sight moving, or feel spinning during the attack?

○ Yes

○ No

1-1 Please specify: heavy/muddled head, stagger or other.

2. How long does a big attack last?

○ No more than 1-2 minutes

○ Dozens of minutes to several hours

○ Several days to several weeks

○ The duration of the attack is not fixed, ranging from several seconds to several days.

○ Symptoms persist without significant remission

3. Is it recurring?

○Yes (if yes, skip to question 3-1)

3-1. Please select the frequency of the attack

Several times a day

Almost every day

Several times a month

Once every few months to several years

The frequency of attacks is not fixed and irregular.

○No

4. How long has it been since the first attack? (Fill in at least one of the following blanks according to your situation)

____ year

____ month

____day

5. Is there any hearing loss?

○Yes

5-1 Please select: Left, right, bilateral, uncertain;

5-2 Please select: sudden decline, gradual decline, uncertain, fluctuating

5-2-1 Please select : fluctuating ，gradually getting worse；fluctuating，getting worse during the attack, but it will get better.

5-3 How long has it been since the initial hearing loss? (Fill in at least one of the following blanks)

____ year

____ month

____day

6. Do you have tinnitus?

○Yes

6-1 Please select: Left, Right, Bilateral, or Uncertain

6-2 whether the tinnitus changes before and after the attack, please select: None, aggravating，aggravating before the attack, alleviating after the attack

○No

7. Do you have ear fullness?

○Yes

7-1 Please select: Left, Right, Bilateral, Uncertain

○No

8. Do you have earache?

○Yes

8-1 Please select: Left, Right, Bilateral, Uncertain

○No

9. Do you experience headache? (multiple answers are allowed)

○ I have a headache during attacks.

○ I had a headache before.

○ I have family history of headache.

○ I have family history of dizziness.

○ None of the above

10.Are you afraid of light or noise, and unwilling to stay in a noisy and overly bright environment?

○Yes

○No

11. Do you have unsteady walking? (multiple answers are allowed)

○Unsteady walking only during attacks

○Unsteady walking without attacks

○Unsteady walking after the onset

○None

12. Do the symptoms get worse while standing or walking?

○Yes

○No

13. Did you fall during the attack?

○Yes

13-1 Please select:

Conscious when falling

Unconscious when falling

○No

14. Did you have unconsciousness or incontinence during the attack?

○Yes

○No

15. Do you have vertigo attacks when lying down, turning over, or getting up quickly from a sitting/lying position?

○Yes

○No

16. Do you have vertigo attacks during breath-holding, exertion, or loud stimulation?

○Yes

○No

17. Are you prone to having vertigo attacks or making symptoms worsen when seeing moving scenes and complicated figure patterns?

○Yes

○No

18. Are you prone to having vertigo attacks after eating certain foods (Such as wine, coffee, tea, chocolate, cheese, preserved food, etc.)

○Yes

○No

19. Are you prone to having vertigo attacks after fatigue, insomnia, or getting angry?

○Yes

○No

20. Do you have upper limb numbness or pain?

○Yes

○No

21. Did you have a cold, fever, vomiting or diarrhea before the onset?

○Yes

○No

22. Have you have a history of ear pus, otitis media, or ear surgery?

○Yes

22-1 Please select: left, right, bilateral, uncertain

○No

23. Have you had a history of head and neck trauma or surgery recently?

○Yes

○No
