# Supplementary material for: A Questionnaire-Based Ensemble Learning Model to Predict the Diagnosis of Vertigo: Model Development and Validation Study
Source: J Med Internet Res. 2022 Aug 3;24(8):e34126. doi: 10.2196/34126 (PMC9386585; doi:10.2196/34126)
Supplement: Multimedia Appendix 2 [file jmir_v24i8e34126_app2.docx]

**Multimedia Appendix 2. Sample size analysis**

It is necessary to verify the requirement of sample size for model development and validation. Thus, we followed Richard D Riley et al[51, 52] to quantify the sufficiency of sample size in terms of the global shrinkage factor $S_{VH}$ given by

$$S_{VH}=1-\frac{p}{LR},$$

in which $p$ is the total number of predictor parameters (ie, $p$=38 in our study). And $LR$ is the likelihood ratio (chi-squared) statistic for the fitted model, ie,

$$LR=-2\left( \ln L_{\mathrm{null}}-\ln L_{\mathrm{model}} \right),$$

where $\ln L_{\mathrm{null}}$ is the likelihood of a model with no predictors, and $\ln L_{\mathrm{model}}$ is that of the fitted model.

The criterion of enough sample size is to ensure a shrinkage factor $S_{VH}$>0.9. Also, given the acceptable shrinkage factor (eg, 0.9), the necessary size of the samples $\underline{n}$ to develop a prediction model that can be estimated, based on the Cox-Snell ratio of explained variance. The results were in Table S1, in which the shrinkage factor satisfies the requirement that $S_{VH}$>0.9 for each of the four diagnostic categories. And our actual sample size has also met the minimal requirement.

**Table S1. The necessary size of the sample size to develop the model.**

| Statistics | BPPV | VM | SSNHL-V | MD |
| --- | --- | --- | --- | --- |
| $\ln L_{\mathrm{null}}$ | -570.2 | -427.6 | -392.6 | -370.0 |
| $\ln L_{\mathrm{model}}$ | -184.3 | -225.7 | -73.3 | -147.9 |
| $LR$ | 771.9 | 403.9 | 638.6 | 444.2 |
| $S_{VH}$ | 95.1% | 90.6% | 94.0% | 91.4% |
| $R_{cs}^{2}$ | 59.8% | 37.9% | 52.9% | 40.8% |
| $\underline{n}$ | 380.8 | 791.2 | 472.1 | 710.3 |

BPPV: benign paroxysmal positional vertigo; VM: vestibular migraine; SSNHL-V: sudden sensorineural hearing loss with vestibular dysfunction; MD: Meniere disease.

Furthermore, modern techniques might be more data-hungry than conventional models like Logistic regression. Thus, we followed van der Ploeg et al[53] to evaluate the sensitivity of our proposed method to sample size. Specifically, we have subsampled the training set for model development from tiny size to hundreds. For each sample size, it has been repeated 30 times to eliminate randomness from sampling. Finally, each model was evaluated in terms of average area under the curve (AUC) on the fixed external set. The results were visualized in Figure S1, in which our proposed method light gradient boosting machine (LGBM) shows robust discrimination performance with subsampled development size, and even dominates the logistic regression. Thus, our proposed method requires less sample size to achieve a given discrimination level than the traditional method Logistic regression, which verifies the sufficiency of sample size for model development again.


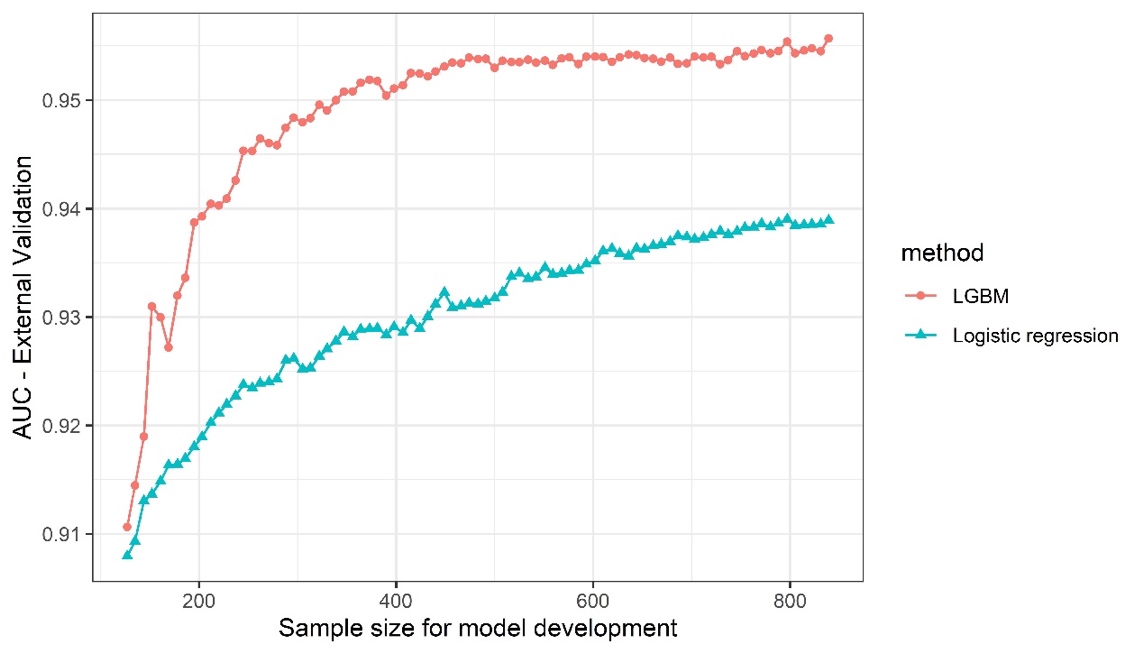


**Figure S1. Compare the sensitivity of LGBM and Logistic regression on sample size.**
